# LL202 protects against dextran sulfate sodium-induced experimental colitis in mice by inhibiting MAPK/AP-1 signaling

**DOI:** 10.18632/oncotarget.11742

**Published:** 2016-08-31

**Authors:** Yuan Gao, Yujie Huang, Yue Zhao, Yang Hu, Zhiyu Li, Qinglong Guo, Kai Zhao, Na Lu

**Affiliations:** ^1^ State Key Laboratory of Natural Medicines, Jiangsu Key Laboratory of Carcinogenesis and Intervention, Jiangsu Key Laboratory of Drug Design and Optimization, China Pharmaceutical University, Nanjing 210009, China; ^2^ School of Pharmacy, China Pharmaceutical University, Nanjing 210009, China

**Keywords:** LL202, colitis, inflammation, AP-1

## Abstract

LL202, a newly-synthesized flavonoid derivative, has been reported to inhibit inflammatory-induced angiogenesis. However, the exact role of LL202 in inflammation along with its mechanism has not been explored. In this study, we investigated the anti-inflammatory effect of LL202 on intestinal inflammation by establishing dextran sulfate sodium (DSS)-induced experimental colitis. LL202 attenuated DSS-induced body weight loss, colon length shortening and colonic pathological damage. The inflammatory cells infiltration, myeloperoxidase (MPO) and inducible nitric oxide synthase (iNOS) activities were decreased by LL202 in a dose-dependent manner. LL202 reduced the production of pro-inflammatory cytokines in serum and colon of DSS-induced mice as well. Mechanically, LL202 could decrease the expression and nuclear translation of AP-1 to protect against DSS-induced colitis. In lipopolysaccharide (LPS)-induced THP-1 cells, LL202 markedly decreased the secretion, mRNA level and protein expression of IL-1β, IL-6 and TNF-α via inhibiting ERK/JNK/p38 MAPK pathways and the nuclear translocation of AP-1. Furthermore, these findings were confirmed in LPS-induced bone marrow derived macrophages (BMDM). In conclusion, our study demonstrated that LL202 could exert its anti-inflammatory effect via inhibiting MAPK/AP-1 signaling, which suggested that LL202 might be a potential effective drug for the treatment of inflammatory bowel diseases.

## INTRODUCTION

Ulcerative colitis (UC) is a kind of intractable complex inflammatory bowel disease (IBD), which is characterized by idiopathic, chronic, relapsing and inflammatory conditions in the gastrointestinal tract [1, 2]. In recent years, the morbidity of UC increases constantly in population and 396 per hundred thousand American individuals suffer from IBD [3]. Although the precise etiology of the disease is enigmatic and unknown, increasing experimental and clinical evidences suggest that a sustained and inappropriate activation of the mucosal immune system with consecutive pro-inflammatory cytokine production plays a crucial role in the development of IBD. Therefore, extensive research is warranted to discover effective anti-inflammatory agents which can inhibit pro-inflammatory cytokine production and ameliorate UC.

The activator protein-1 (AP-1) transcription factor has represented a paradigm for gene regulation implicated in chronic inflammatory diseases [4, 5]. AP-1 consists of a variety of hetero- or homo-dimeric complex and the dimers of Jun and Fos are translocated to the nucleus and initiate the transcription of downstream target genes [6, 7]. Mitogen-activated protein kinases (MAPK) are composed of three well-characterized subfamilies, including extracellular signal-regulated kinases (ERK1/2), Jun N-terminal kinases (JNK) and p38, which are responsible for the expression and activation of AP-1 [8, 9]. Lipopolysaccharide (LPS) is thought to be the major pathogenic factor involved in inflammation [10]. The previous studies have reported that LPS enhanced the expression and nuclear transcription of AP-1 [11, 12]. In addition, pro-inflammatory cytokines are also act as important target genes of c-Jun and c-Fos [13, 14], among which IL-1β, IL-6 and TNF-α play an indispensable role in the development of ulcerative colitis [1]. The pro-inflammatory pathogenic role of AP-1 implies that inhibition of MAPK/AP-1 signaling is a promising strategy for IBD therapy.

Various kinds of flavonoids have been reported to exert anti-inflammatory properties, including wogonin, baicalein, quercetin and so on [15–17]. LL202 is a newly-synthesized flavonoid, which bears the three-ring structure of the flavone backbone and possesses a strong ability to inhibit LPS-induced angiogenesis [18]. However, whether LL202 could affect LPS-induced pro-inflammatory cytokines production and inflammatory diseases such as colitis, along with the molecular mechanisms, remain unknown and warrant further investigations.

In our study, we investigated the anti-inflammatory effect of LL202 on intestinal inflammation and the potential mechanisms. We established the DSS-induced experimental colitis to evaluate the protective effect of LL202 on inflammation *in vivo*. Further mechanism researches revealed that LL202 protected against DSS-induced colitis by inhibiting the expression and nuclear translocation of AP-1 in colons. In addition, these findings were confirmed in LPS-induced THP-1 cells and bone marrow derived macrophages (BMDM) *in vitro*. Taken together, these results suggested that LL202 prevented DSS-induced colitis through a potential mechanism attributed to inhibiting MAPK/AP-1 signaling and LL202 may serve as a candidate in the treatment of colitis.

## RESULTS

### LL202 ameliorated colon injury and inflammatory symptoms in DSS-induced colitis in mice

**Figure 1:**
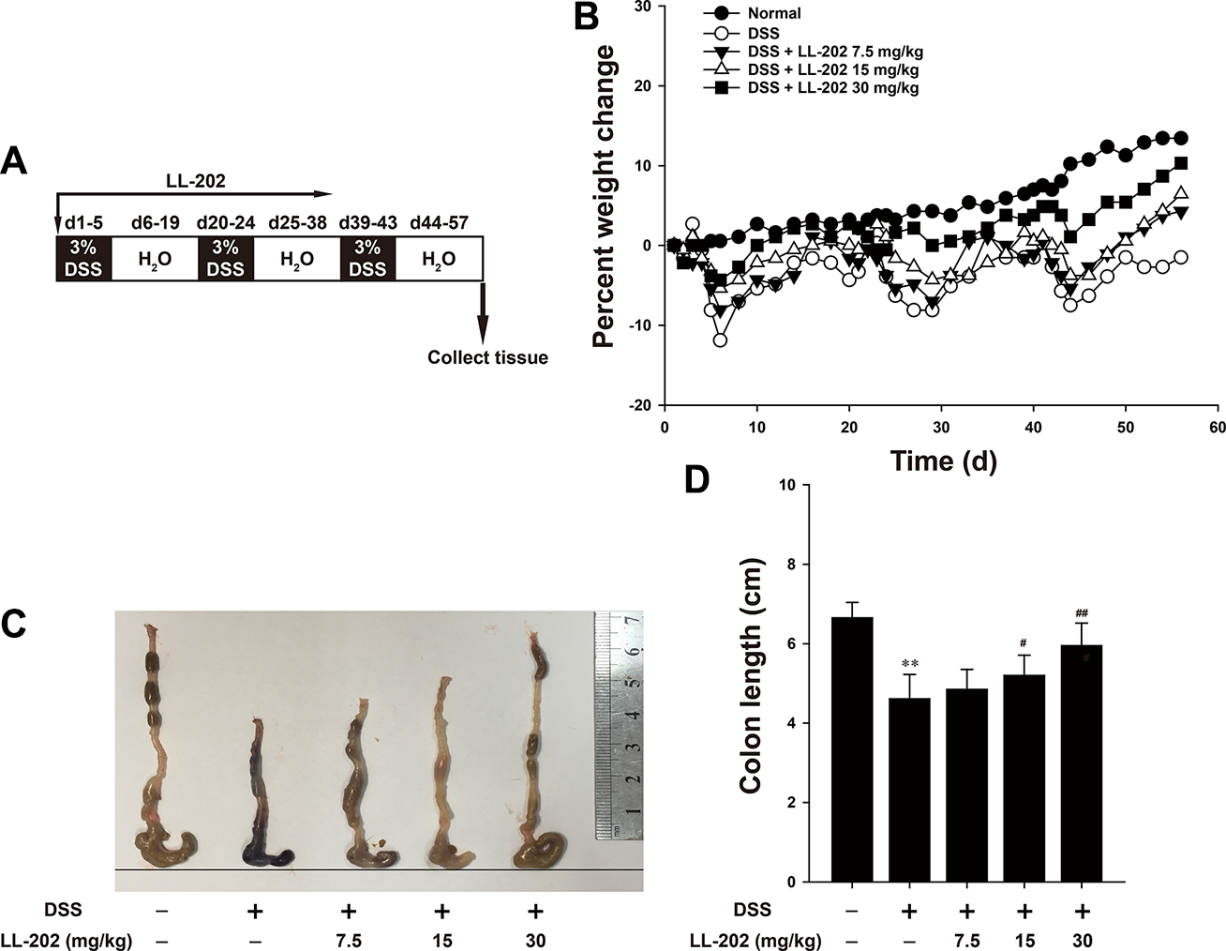
LL202 reduced the susceptibility of mice to DSS-induced experimental colitis C57BL/6 mice were subjected to a DSS-induced colitis induction protocol using three cycles of 3% DSS in drinking water. (**A**). Diagram shows the experimental course of DSS-induced colitis mouse model. (**B**). Body weights of each group (*n* = 8 per group) were measured after DSS induction of colitis. (**C**–**D**). Macroscopic appearance and quantification of the length of colons from each group of mice were carried out. Each experiment was performed at least three times. Data are presented as mean ± SD. *p < 0.05, **p < 0.01 compared with normal mice; ^#^*p* < 0.05, ^##^*p* < 0.01 compared with DSS-treated colitis mice.

To investigate the effects of LL202 on colitis development, we established the DSS-induces model in C57BL/6 mice (Figure 1A), which is a well-established preclinical model that exhibits many phenotypic features related to human ulcerative colitis [19]. An obvious character of DSS-induced colitis is the significant loss of body weight, so body weights of mice were monitored throughout the study. The results showed that animals lost weight after DSS treatment, but LL202 increased the body weight of DSS-treated colitis mice significantly (Figure 1B). It has been reported that DSS-induced colon shortening is a crucial marker of colitis. As shown in Figure 1C and 1D, the colon was obviously longer in LL202-treated mice than in DSS-treated mice.

**Figure 2:**
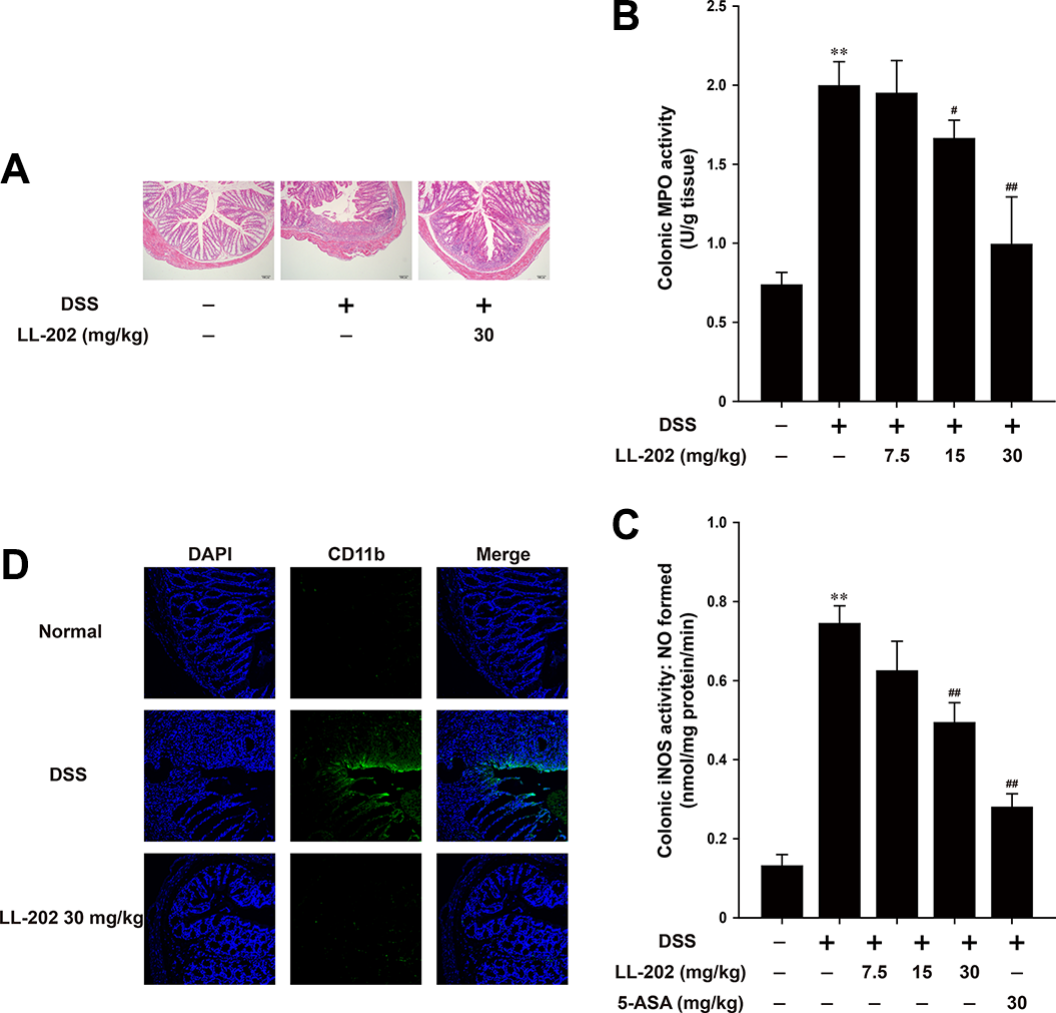
LL202 protected against DSS-induced colon damage in mice (**A**). Serial sections of colon tissues were stained with hematoxylin and eosin (H&E). (**B**–**C**). MPO and iNOS activities in the colonic tissues were detected. (**D**). Sections of colon tissues were immunostained with DAPI (blue) and anti CD11b-FITC (green) and observed by confocal laser-scanning microscope. Each experiment was performed at least three times. Data are presented as means ± SD. *p < 0.05, **p < 0.01 compared with normal mice; ^#^*p* < 0.05, ^##^*p* < 0.01 compared with DSS-treated colitis mice.

We next performed histopathological analysis using Haematoxylin & Eosin (H&E) staining (Figure 2A) to verify the protective effect of LL202 on colitis. The mucosal damage was observed apparently in DSS-treated mice, which is characterized by ulceration accompany massive granulocytes and mononuclear cells infiltration into the mucosa as well as congestion and edema of the submucosa. However, LL202 (30 mg/kg) maintain intact colonic architecture with no obvious ulcer and inhibited inflammatory cell infiltration. Moreover, LL202 suppressed DSS-induced myeloperoxidase (MPO) and inducible nitric oxide synthase (iNOS) activities notably (Figure 2B and 2C). CD11b is expressed on the surface of various kinds of leukocytes [20], which can be used as an indicator to monitor the occurrence of inflammation. As shown in Figure 2D, a great number of CD11b^+^ inflammatory cells accumulated at the mucosa of the lesion site in colonic tissues from DSS-treated mice were presented obviously. LL202 (30 mg/kg) could reduce the number of infiltrating CD11b^+^ inflammatory cells in colon tissues. Taken together, these results indicated that LL202 could ameliorate DSS-induced colitis.

### LL202 inhibited the infiltration of pro-inflammatory cytokines in DSS-induced colitis mice

**Figure 3:**
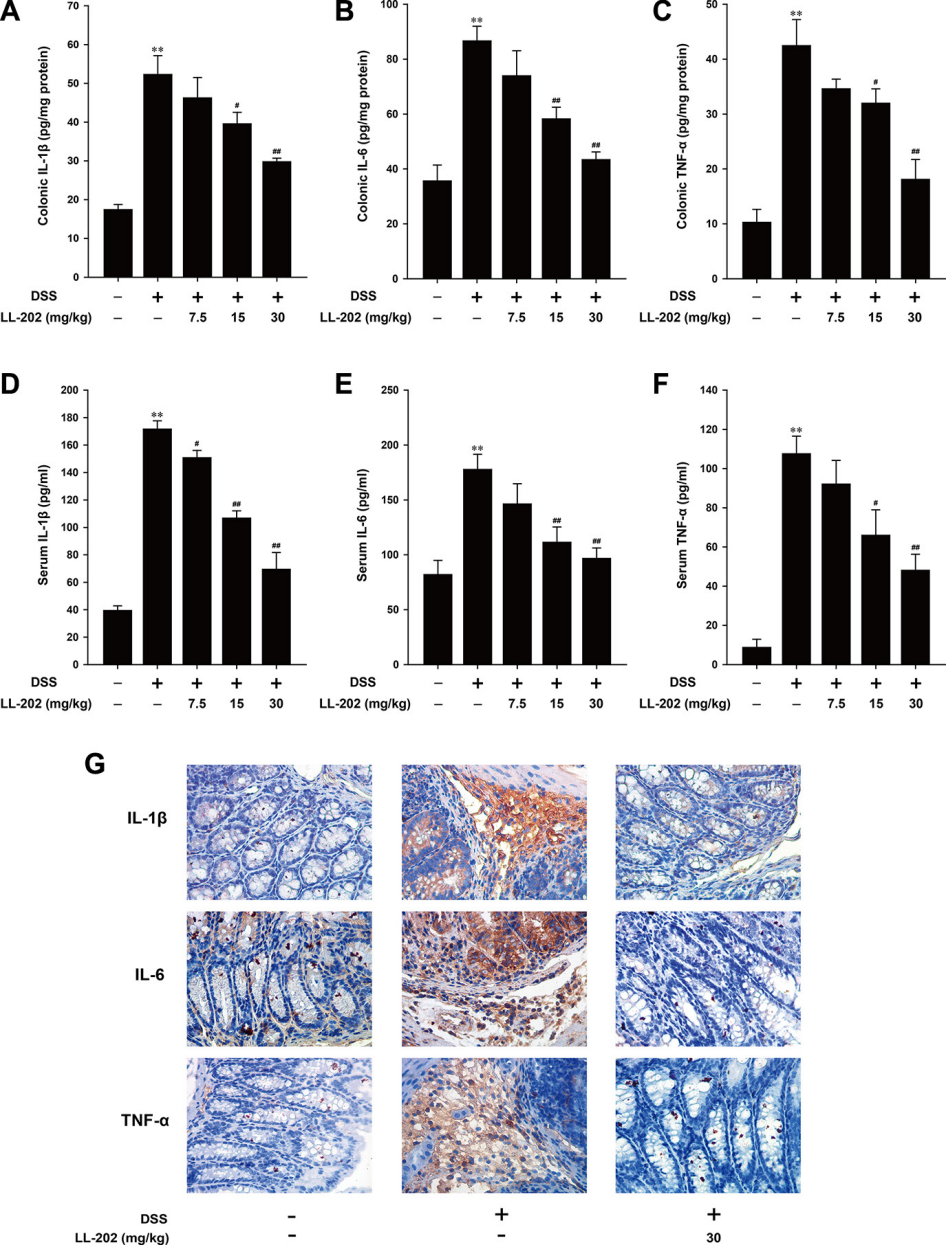
LL202 inhibited pro-inflammatory cytokines production in colon tissues and serum of DSS-colitis mice (**A**–**C**) The secretion of inflammation-related cytokines IL-1β, IL-6 and TNF-α in colonic homogenate was tested by ELISA. (**D**–**F**) The production of IL-1β, IL-6 and TNF-α in serum was determined by ELISA. (**G**) Expression of IL-1β, IL-6 and TNF-α was detected by immunohistochemistry in colonic tissues (×40). Each experiment was performed at least three times. Data were presented as means ± SD. *p < 0.05, **p < 0.01 compared with normal mice; ^#^*p* < 0.05, ^##^*p* < 0.01 compared with DSS-treated colitis mice.

Increased secretion and its infiltration of pro-inflammatory cytokines play a key role in the development of DSS-induced colitis [21, 22]. Therefore, we assessed the level of several main inflammatory cytokines to gain an insight into the effect of LL202 on the inflammatory status of DSS-induced colitis. As shown in Figure 3A–3C, the secretory level of IL-1β, IL-6 and TNF-α in colonic homogenates was significantly increased after DSS challenge, while LL202 inhibited the elevated secretion of these cytokines in a dose-dependent manner. We then measured the level of IL-1β, IL-6 and TNF-α in the serum. The DSS-induced high content of these pro-inflammatory cytokines in serum was remarkably suppressed by LL202 (Figure 3D–3F). In addition, LL202 at 30 mg/kg markedly reduced the infiltration of IL-1β, IL-6 and TNF-α-positive inflammatory cells in colonic mucosa of DSS-induced mice (Figure 3G).

### LL202 suppressed DSS-induced AP-1 signaling *in vivo*

**Figure 4:**
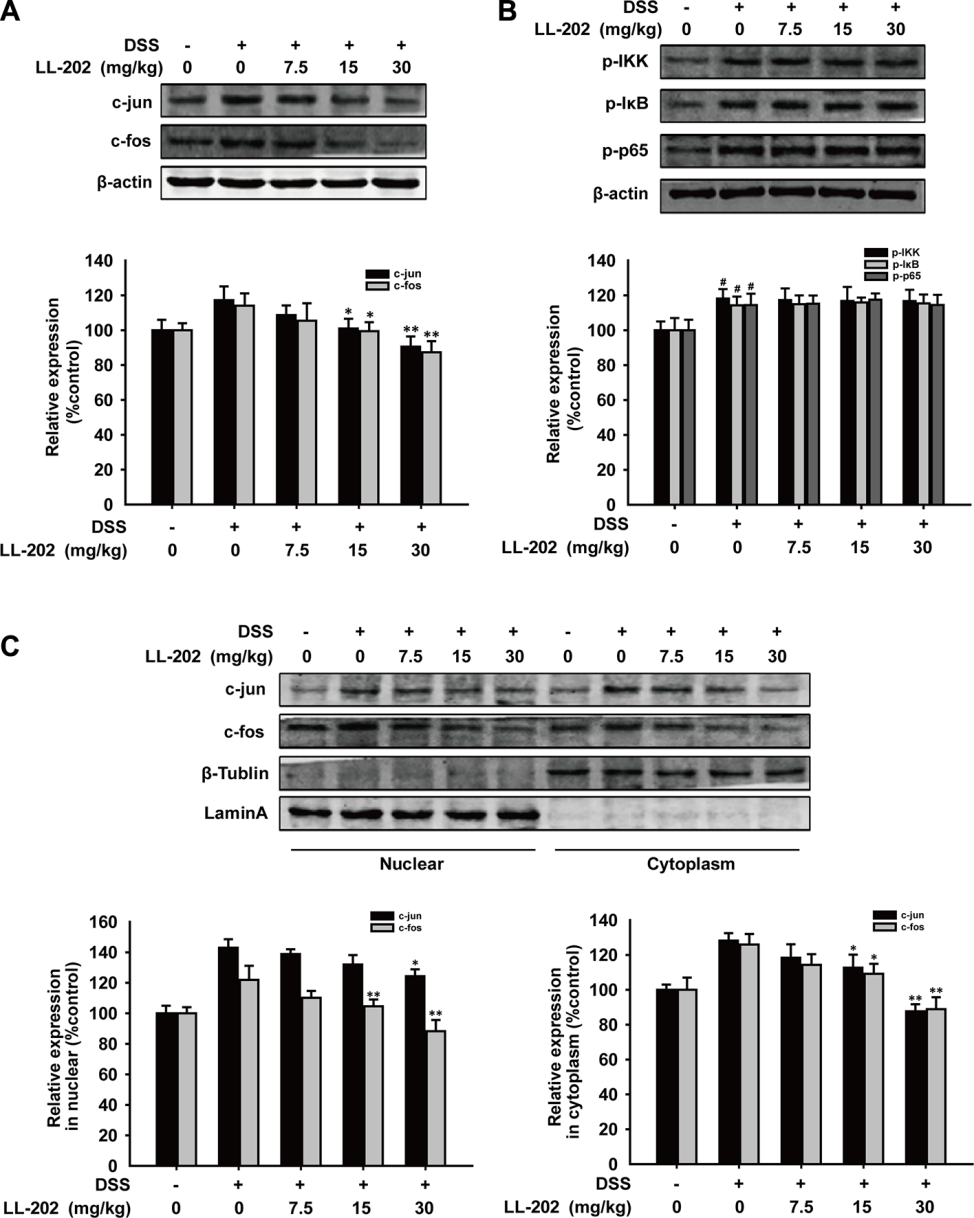
Effects of LL202 on AP-1 and NF-κB signaling in colon tissues of DSS-colitis mice (**A**) Total protein expression of c-Jun and c-Fos in colonic tissues were detected by Western Blot. (**B**) Phosphorylation of IKK, IκB and p65 in colonic homogenate from each group of mice were subjected to Western Blot using specific antibodies. β-actin was used as an internal control. (**C**) Nuclear translocation of c-Jun and c-Fos in colonic tissues were determined by Western Blot. Densitometric analysis was performed to determine the relative ratios of each protein. Lamin A and β-Tublin were used as nuclear and cytoplasmic markers, respectively. The results were representative of three independent experiments. Data were presented as means ± SD. ^##^*P* < 0.01 compared with normal mice; *P < 0.05, **P < 0.01 compared with DSS-treated colitis mice.

As AP-1 and NF-κB are two important transcriptional regulators of these pro-inflammatory cytokines [23, 24], we sought to determine whether LL202 could inhibit AP-1 or NF-κB signaling. As shown in Figure 4A, LL202 remarkably down-regulated the expression of c-Jun and c-Fos in the colon tissues of DSS-induced colitis mice. In addition, compared to the control group, LL202 decreased the expression level of c-Jun and c-Fos in both cytosolic and cell nucleus (Figure 4C). However, LL202 showed little effect on the increased expression of p-IKK, p-IκB and p-p65. These results suggested that LL202 could inhibit the expression and nuclear translocation of c-Jun and c-Fos without affecting NF-κB signaling in DSS-induced colitis mice.

### LL202 inhibited pro-inflammatory cytokines production *in vitro*

**Figure 5:**
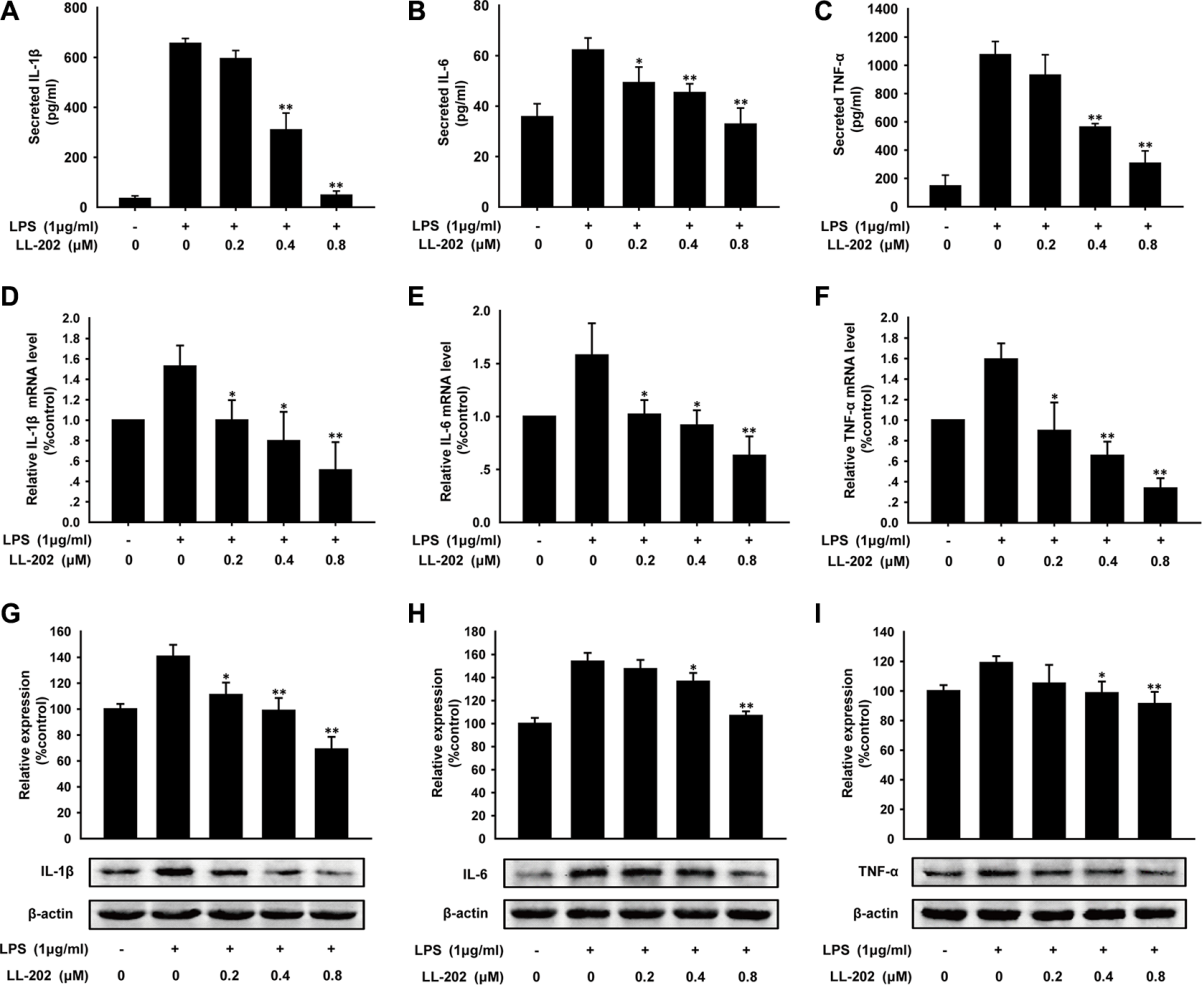
LL202 inhibited the secretion, mRNA and protein level of pro-inflammatory cytokines in LPS-induced THP-1 cells THP-1 cells were treated with LPS (1 μg/ml) and the indicated concentrations of LL202 for 6 h. (**A**) Secretion of IL-1β, IL-6 and TNF-α in the cell supernatant were analyzed by ELISA. (**B**) The mRNA level of IL-1β, IL-6 and TNF-α were measured by real-time RT-PCR. (**C**) Protein expression of IL-1β, IL-6 and TNF-α were detected by Western Blot with densitometric analysis to determine the relative ratio normalized to β-actin. The results are representative of three independent experiments and expressed as means ± SD. *P < 0.05, **P < 0.01 compared with LPS-treated group.

It was reported that IL-1β, IL-6 and TNF-α play key roles in inflammation-related diseases [25, 26]. To confirm our conclusion that LL202 inhibited the secretion of IL-1β, IL-6 and TNF-α *in vivo*, we investigated the effect of LL202 on LPS-induced THP-1 cells. As expected, the result showed that the secretion of IL-1β, IL-6 and TNF-α induced by LPS was reduced by LL202 in a concentration-dependent manner (Figure 5A–5C). Furthermore, we found that the mRNA (Figure 5D–5F) and protein (Figure 5G–5I) level of IL-1β, IL-6 and TNF-α were also increased in LPS-stimulated THP-1 cells, while the increased expression was reversed by LL202 treatment. Taken together, all of these findings demonstrated that LL202 inhibited the expression and secretion of IL-1β, IL-6 and TNF-α.

### LL202 inhibited the MAPK/AP-1 signaling *in vitro*

**Figure 6:**
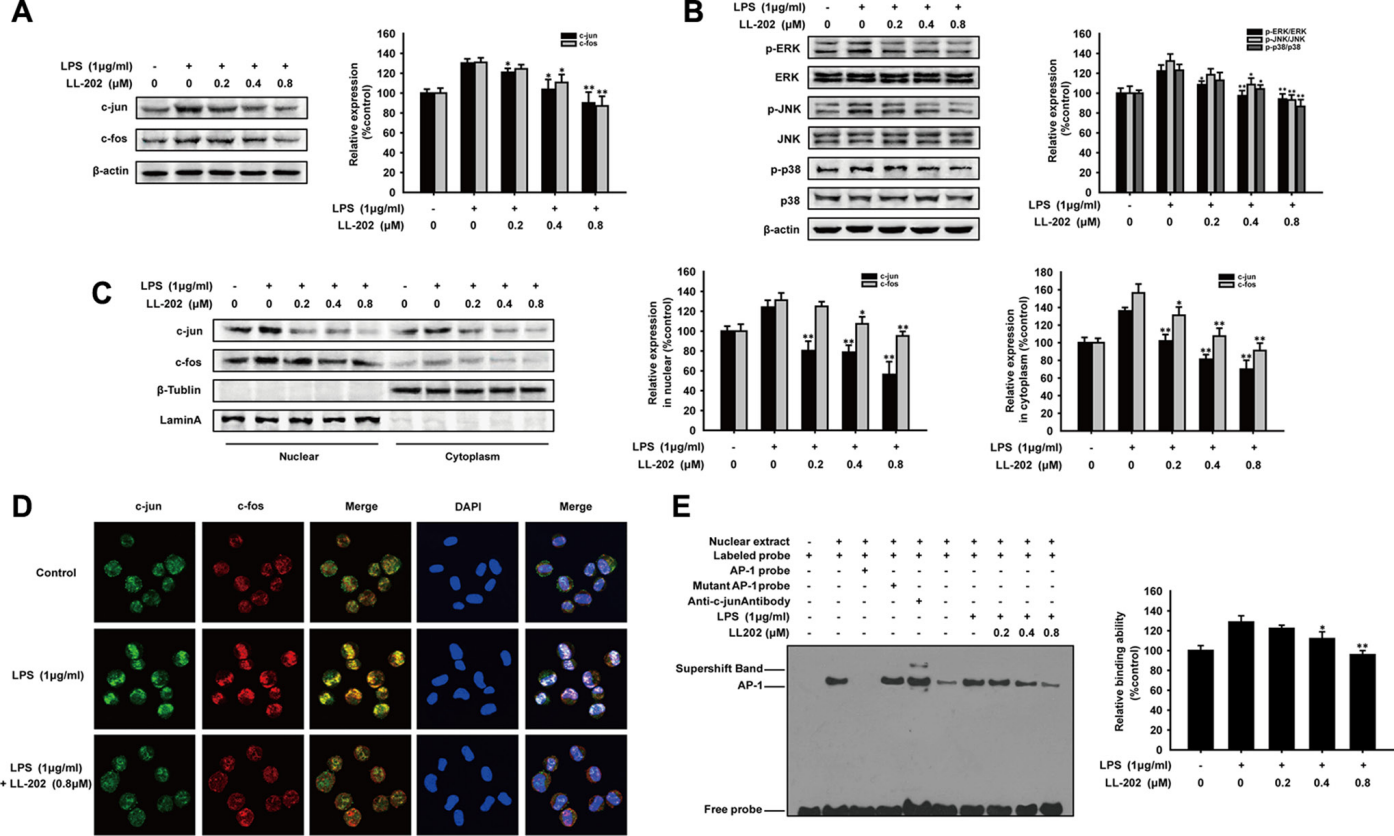
LL202 inhibited MAPK/AP-1 signaling in LPS-induced THP-1 cells THP-1 cells were treated with LPS (1 μg/ml) and the indicated concentrations of LL202 for 6 h. (**A**) Total Protein expression of c-Jun and c-Fos were detected by Western Blot. (**B**) Effect of LL202 on the expression of p-ERK, ERK, p-JNK, JNK, p38 and p-p38 was detected by Western blot analysis using specific antibodies. β-actin was used as an internal control. (**C**) Nuclear and cytoplasmic protein level of c-Jun and c-Fos in LPS-stimulated THP-1 cells were determined by Western Blot. (**D**) Immunofluorescence was performed to analyze the nuclear translocation of c-Jun and c-Fos. (**E**) Nuclear extracts were prepared and subjected to EMSA to detect DNA-binding activity of AP-1. The results are representative of three independent experiments and expressed as means ± SD. *P < 0.05, **P < 0.01 compared with LPS group.

To elucidate the anti-inflammatory mechanism of LL202 *in vitro*, we examined the effect of LL202 on the expression of AP-1 in LPS-induced THP-1 cells. As expected, LL202 down-regulated the protein level of c-Jun and c-Fos noticeably in THP-1 cells stimulated by LPS (Figure 6A). Meanwhile, we observed that c-Jun or c-Fos in cytosolic and cell nucleus were both decreased by western blot analysis. Immunofluorescence showed that the nuclear translocation and the interaction of c-Jun and c-Fos were decreased by LL202 (0.8 μM). The previous study has shown that MAPK are responsible for the expression, activation and nuclear translocation of Jun and Fos proteins [27]. We then determined whether LL202 exerted the inhibition effect to the activation of ERK/JNK/p38 MAPK pathways. Our results showed that LL202 markedly suppressed LPS-induced phosphorylation of JNK, ERK1/2 and p38 (Figure 6B). In addition, EMSA analysis further demonstrated that LL202 decreased the DNA binding activity of AP-1 in THP-1 cells (Figure 6E). On the basis of these findings, we proposed that LL202 might exert an anti-inflammatory effect by inhibiting MAPK/AP-1 signaling.

### LL202 reduced pro-inflammatory cytokines production in IL-6 or TNF-α stimulated THP-1 cells

**Figure 7:**
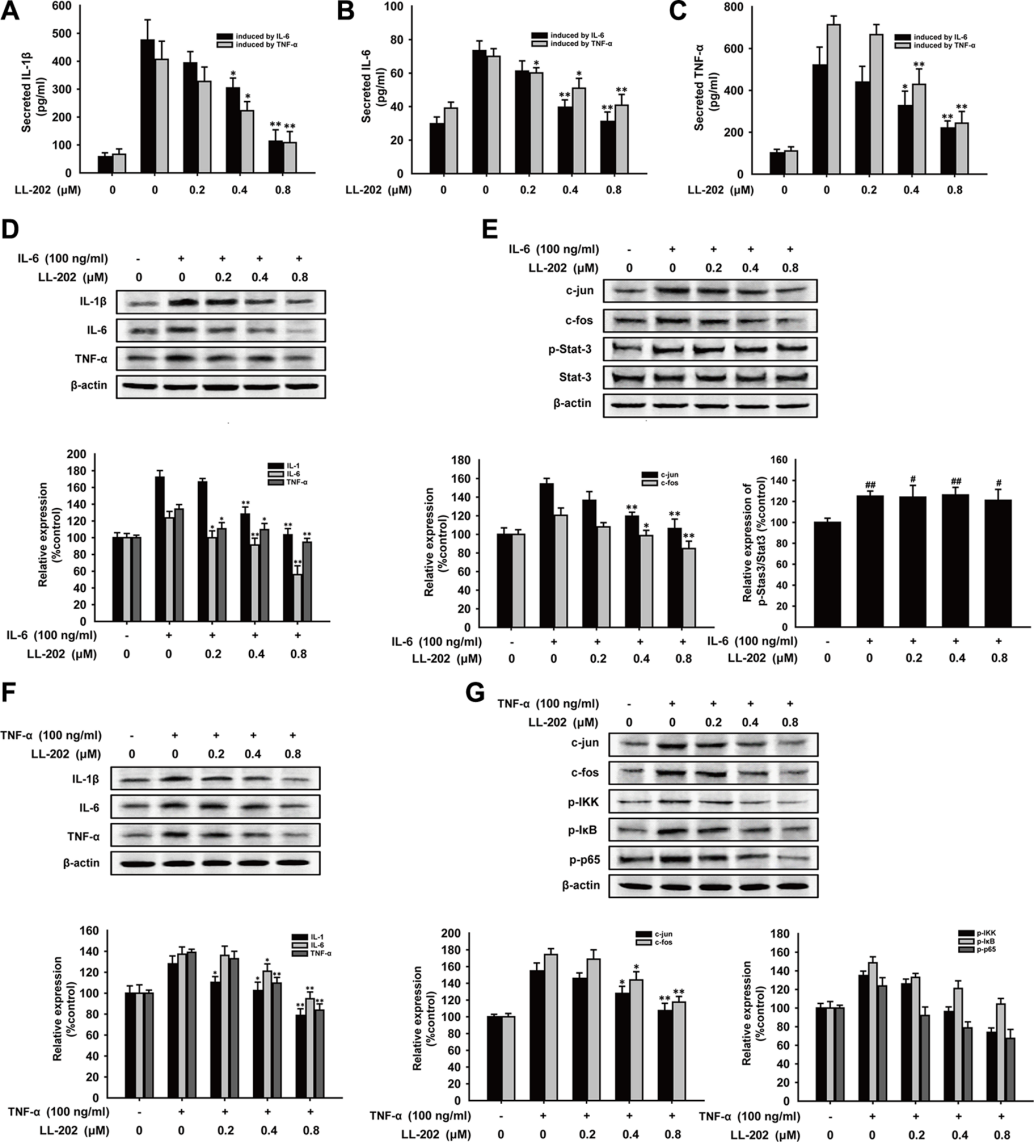
Effects of LL202 on the production of pro-inflammatory cytokines and AP-1 signaling in IL-6 or TNF-α stimulated THP-1 cells THP-1 cells were stimulated with IL-6 (100 μg/ml) or TNF-α (100 μg/ml) alone for 12 h and then treated with the indicated concentrations of LL202 for another 6 h. (**A**–**C**) Secretion of IL-1β, IL-6 and TNF-α in the cell supernatant were analyzed by ELISA. (**D**) Protein expression of IL-1β, IL-6 and TNF-α in IL-6 stimulated THP-1 cells were detected by Western Blot. (**E**). Effect of LL202 on the expression of c-Jun, c-Fos, p-Stat-3 and Stat-3 in IL-6-induced THP-1 cells was detected by Western blot analysis. (**F**). Protein expression of IL-1β, IL-6 and TNF-α in TNF-α stimulated THP-1 cells were detected by Western Blot. (**G**). Effect of LL202 on the expression of c-Jun, c-Fos, p-IKK, p-IκB and p-p65 in TNF-α-induced THP-1 cells was detected by Western blot analysis using specific antibodies. β-actin was used as an internal control. The results were representative of three independent experiments. Data were presented as means ± SD. ^##^*P* < 0.01 compared with normal mice; *P < 0.05, **P < 0.01 compared with DSS-treated colitis mice.

To further demonstrate the anti-inflammation effect of LL202, we tested the secretion and expression of IL-1β, IL-6 and TNF-α in THP-1 cells stimulated by adding IL-6 or TNF-α, respectively. THP-1 cells were rinsed with PBS to remove the exogenous pro-inflammatory cytokines after stimulation with IL-6 (100 ng/ml) or TNF-α (100 ng/ml) alone for 12 h, and then treated with indicated concentrations of LL202 for another 6 h. ELISA showed that the concentration of IL-1β, IL-6 and TNF-α was decreased dramatically in the supernatant of THP-1 cells stimulated by IL-6 or TNF-α (Figure 7A–7C). Consistently, LL202 could also reduce the expression of IL-1β, IL-6 and TNF-α in THP-1 cells stimulated by exogenous IL-6 or TNF-α in a concentration-dependent manner (Figure 7D and 7F). In further detection of inactivation of the related-signaling, we found that LL202 could inhibit the activation of AP-1 and NF-κB signaling induced by IL-6 or TNF-α, while had no effect on the activator of transcription 3 (STAT3)signaling in IL-6 stimulated THP-1cells (Figure 7E and 7G). Taken together, LL202 could inhibit AP-1 and NF-κB without affecting IL-6/STAT3 signaling *in vitro*.

### LL202 inhibited pro-inflammatory cytokines production via inhibiting AP-1 signaling in BMDMs

**Figure 8:**
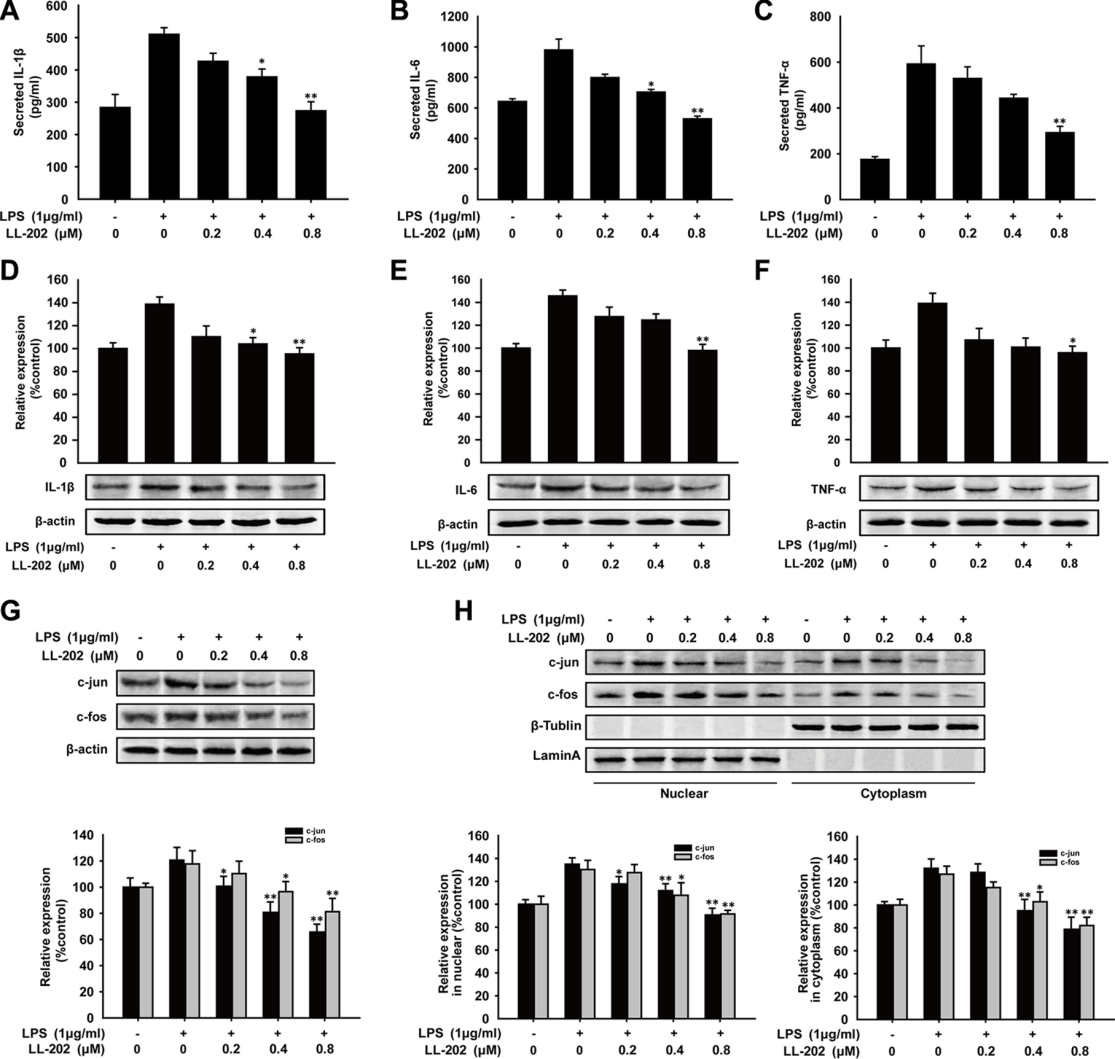
Effects of LL202 on pro-inflammatory cytokines production and AP-1 expression in LPS-induced BMDMs BMDMs were treated with LPS (1 μg/ml) and the indicated concentrations of LL202 for 6 h. (**A**) Secretion of IL-1β, IL-6 and TNF-α in the cell supernatant were analyzed by ELISA. (**B**) Expression of IL-1β, IL-6 and TNF-α were detected by Western Blot with densitometric analysis to determine the relative ratio normalized to β-actin. (**C**) Expression of c-Jun and c-Fos were detected by Western Blot. (**D**) The separation of nuclear and cytoplasmic protein in BMDMs was performed. Nuclear and cytoplasmic protein level of c-Jun and c-Fos in colonic tissues were determined by Western Blot using specific antibodies. Densitometric analysis was performed to determine the relative ratios of each protein. Lamin A and β-Tublin were used as nuclear and cytoplasmic markers, respectively. The results are representative of three independent experiments and expressed as means ± SD. *P < 0.05, **P < 0.01 compared with LPS group.

To further investigate the anti-inflammatory effect of LL202, we treated the bone marrow derived macrophages (BMDM) isolated from C57BL/6 mice with indicated concentration of LL202. As shown in Figure 8A–8F, LL202 decreased LPS-induced secretion and expression of IL-1β, IL-6 and TNF-α in BMDMs. In addition, the expression level and nuclear translocation of c-Jun and c-Fos were both reduced by LL202 in a concentration-dependent manner (Figure 8G and 8H). These findings above indicated that LL202 might exert an anti-inflammatory effect via inhibiting AP-1 in BMDMs.

## DISCUSSION

Ulcerative colitis, a prevalent inflammatory bowel disease in the developed countries, is becoming more and more common in developing countries these days [28, 29]. Recently, there are various emerging therapeutic drugs for UC, such as sulfasalazine, azathioprine, corticosteroids, classical immunosuppressants and TNF-α antibody [30, 31]. However, most of these drugs have limitations in efficacy and wide clinical applications for their serious side effects [32, 33]. Hence, novel effective therapeutic agents with fewer side effects and wider application are urgent to be developed.

The model of DSS-induced colitis in mice, which shows the similar pathological features with human ulcerative colitis, has been widely used in lots of studies related to human inflammatory bowel disease [34]. Here, we performed this model to evaluate the protective effect of LL202 against colitis. LL202 attenuated the serious intestinal injury and inflammatory symptoms in DSS-induced colitis mice, including body weight loss, colon length shortening and colonic tissue damage. The activity of MPO is a marker of neutrophil infiltration [35] and iNOS can destroy the mucosa and submucosa of the intestine by regulating NO release [36], both of which can be considered as index of inflammation damage. In DSS-induced colitis mice, LL202 decreased MPO and iNOS activities, which suggested that LL202 could reverse the infiltration of inflammatory cells into colon tissues (Figure 2B–2D).

The high level of pro-inflammatory cytokine secretion and expression is a hallmark of DSS-induced colitis [21, 37]. It has been reported that the overabundance of IL-1β, IL-6 and TNF-α plays incomparable role in promoting inflammation development [38–40]. LL202 inhibited the high-production of IL-1β, IL-6 and TNF-α dramatically both in the colon and serum of DSS-induced colitis mice. In addition, the similar inhibitory effects of LL202 on the production of pro-inflammatory cytokines were also observed in THP-1 cells and BMDMs *in vitro*. Thus, LL202 could protect against DSS-induced colitis by inhibiting the production of pro-inflammatory cytokines.

LPS is commonly used as a potent inflammatory stimulus, recognized by its sensor, toll-like receptors 4 (TLR4), and then initiate the downstream singling to trigger the expression of pro-inflammatory cytokines [41]. MAPK, is one of the downstream pathways, which leads to the induction of two main transcription factors AP-1 and NF-κB. They both control the expression of pro-inflammatory cytokines including IL-1β, IL-6 and TNF-α [42]. The previous study showed that LL202 can modulate LPS-induced angiogenesis by decreasing the expression of TLR4 and inhibiting the activation of MAPK and NF-κB pathways [18]. Therefore, we hypothesized that the inhibition of AP-1 and NF-κB nuclear translocation may account for the underlying molecular mechanism of LL202 that inhibited the production of pro-inflammatory cytokines. As a result, we found that LL202 could inhibit the expression and nuclear translocation of AP-1 without affecting NF-κB signaling in DSS-induced colitis mice. These results were further confirmed in LPS-induced THP-1 cells and BMDMs *in vitro*, and the nuclear translocation and binding with DNA of AP-1 were also inhibited by LL202 in LPS-induced THP-1cells (Figure 6D and 6E).

To further demonstrate the inhibitory effect of LL202 on AP-1, THP-1 cells we treated with IL-6 and TNF-α, another two pro-inflammatory cytokines, respectively. IL-6 binds to the IL-6 receptor complex, activates JAK/STAT3 signaling and then phosphorylated-STAT3 (p-STAT3) translocates into the nucleus to promote the transcription of its target genes [43]. However, LL202 could inhibit the increased expression of c-Jun, c-Fos induced by IL-6 without affecting the activation of STAT3. It indicated that LL202 could not be a broad-spectrum inhibitor of phosphorylation. As NF-κB could be simultaneously activated by TNF-α [44], we detected the effect of LL202 on NF-κB signaling in TNF-α stimulated THP-1 cells. Phosphorylation of IKK, IκB and p65 were inhibited by LL202 in a concentration-dependent manner. These results of mechanism research were supported by the previous study that LL202 inhibited the activation of MAPK and NF-κB pathways in LPS-induced HUVECs and that TLR4 signaling might serve as the upstream target [18]. However, it was strange that LL202 could not inhibit NF-κB signaling in DSS-induced colitis mice, but blocked NF-κB pathway activated by TNF-α *in vitro*. Yet the activation of AP-1 both *in vivo* and *in vitro* was suppressed by LL202 apparently. It might be on account of the difference of LL202 between *in vivo* and *in vitro*. These specific effects of LL202 will be studied in the future research.

In conclusion, we demonstrated that LL202 could protect against DSS-induced colitis in mice by inhibiting MAPK/AP-1 signaling for the first time. These findings provided the evidence that LL202 could be developed as a therapeutic agent for colitis and have advantages for potential clinical applications in the future.

## MATERIALS AND METHODS

### Reagents and antibodies

LL-202 (C_25_H_29_BrN_2_O_5_, MW:517.41), prepared from Dr. Zhiyu Li (China Pharmaceutical University, China), was dissolved in 100% dimethyl sulfoxide (DMSO) and stored at −20°C. *In vivo* study, LL-202 was prepared as intragastric administration (0.5% sodium carboxyl methyl cellulose) by Dr. Xue Ke from College of Pharmacy, China Pharmaceutical University.

Dimethylsulfoxide (DMSO) and LPS (*E.coli*: Serotype 055:B5) were purchased from Sigma Chemical Co. (St. Louis, MO). Dextran sulfate sodium (DSS) was obtained from MP Biomedicals Inc. (Irvine, CA, USA). CMC was obtained from Sinopharm Group Co. Ltd. (Shanghai, China). Paraformaldehyde (PFA) was purchased from Yonghua Chemical Technology Co. Ltd. (Changshu, China). Dye DAPI was purchased from Invitrogen (Carlsbad, CA, USA). Triton X-100 was purchased from Chao Rui Biotech. Co. Ltd. (Shanghai, China). BSA was purchased from Roche Diagnosis Ltd. (Basel, Switzerland).

Myeloperoxidase (MPO) activity assay kit was purchased from Jiancheng Bioengineering Institute (Nanjing, China). Nitric Oxide Synthase (NOS) Assay Kit was purchased from Beyotime Institute of Biotechnology (Nanjing, China). ELISA kits for mouse IL-1β, IL-6, TNF-α and human IL-1β, IL-6, TNF-α were purchased from Boster Biotech Co. Ltd. (Wuhan, China).

Primary antibodies against IL-1β, IL-6, TNF-α, c-Fos, and β-actin were obtained from Santa Cruz Biotechnology (Santa Cruz, CA, USA). Antibodies against c-Jun, p-STAT3, STAT3 and Lamin A were purchased from Bioworld (Bioworld, Minnesota). Anti-CD11b was purchased from eBioscience (San Diego, CA, USA). IRDyeTM800 conjugated secondary antibodies were obtained from Rockland Inc. (Philadelphia, PA, USA).

### Cell culture

Human acute monocytic leukemia THP-1 cells were obtained from Cell Bank of the Chinese Academic of Sciences (Shanghai, China). THP-1 cells were cultured in RPMI-1640 medium (Gibco, Carlsbad, CA, USA), supplemented with 10% fetal bovine serum (Gibco, CA, USA), 100 U/ml benzyl penicillin and 100 mg/ml streptomycin. Cells were cultured in a humidified environment with 5% CO_2_ at 37°C. Bone marrow derived macrophages (BMDM) were isolated from C57BL/6 mice and cultured with DMEM supplemented with 10% fetal bovine serum and 20 ng/ml GM-CSF (PeproTech, USA).

### DSS-induced colitis and design of drug treatment

Six to eight weeks old female C57BL/6 mice, weighing 18–22 g, were supplied by Shanghai Laboratory Animal Center, China Academy of Sciences. Experimental protocols were in accordance with National Institutes of Health regulations and approved by the Institutional Animal Care and Use Committee. Throughout the acclimatization and study periods, all animals had access to food and water *ad libitum* and were maintained on a 12 h light/dark cycle (21 ± 2°C with a relative humidity of 45 ± 10%).

Chronic colitis was induced by administration of DSS in drinking water. The mice were received either drinking regular water (control) or 3% (w/v) DSS drinking water (model) for 5 days. After this, mice were maintained on regular water for 14 days and subjected to two more DSS treatment cycles. The mice were randomly assigned to control, DSS-treated and LL202 (7.5, 15 or 30 mg/kg)-treated groups. LL202 were given by oral gavage every day from the first day to the termination of the experiment, respectively.

### Macroscopic assessment and histological analysis of colonic lesions

Animals were weighed and inspected daily. After colitis induction animals were sacrificed, colons were removed, opened longitudinally, and washed with phosphate-buffered saline (PBS) and pieces of colonic tissue were used for *ex vivo* analysis. The histological analysis was performed as previously described [46].

### Assessment of myeloperoxidase (MPO) activity

Neutrophil infiltration into inflamed colonic mucosa was quantified by MPO activity assessment using the O-dianisidine method. Proteins extracted from colonic tissues were used to assess MPO levels according to manufacturer's instructions.

### Measurement of iNOS activity

The supernatant of colonic tissue was measured by Nitric Oxide Synthase Assay Kit according to the manufacturer's recommendations.

### Immunofluorescence of CD11b in colon tissues

CD11b positive inflammatory cell infiltration analysis was performed on paraffin-embedded colon tissue sections. Briefly, the sections were deparaffinized, rehydrated and washed in 1% PBS Tween. Then they were treated with 3% hydrogen peroxide, blocked with 3% bovine serum albumin (BSA) and incubated for 1 h at room temperature with anti-CD11b FITC (1:100). The slides were then counter-stained with DAPI for 30 min. The reaction was stopped by thorough washing in water for 5 min. Images were acquired by confocal laser-scanning microscope (Olympus, Tokyo, JP).

### Cytokine quantification by enzyme-linked immunoassay (ELISA)

Colons from mice in each group were homogenated with lysis buffer to extract total protein. The homogenate was centrifuged at 12,000 rpm at 4°C for 15 min. The amount of total extracted protein was determined by BCA TM protein assay kit (Thermo, MA, USA). The amounts of IL-1β, IL-6 and TNF-α in the colon homogenate were measured by ELISA kit. IL-1β, IL-6 and TNF-α production in supernatant of THP-1 cells, BMDMs and serum of mice were measured by ELISA kits according to the manufacturers' recommendations.

### Quantitative real-time PCR analysis

Total RNA was isolated with Tripure Isolation Reagent (Roche, Mannheim, Germany). The real-time PCR (RT-PCR) kit was purchased from TaKaRa Biotechnology Co. Ltd. (Dalian, China). Reactions were conducted according to the instructions. Samples were run on the ABI 7500 Real-Time PCR system. Each reaction was done in triplicate, and the threshold values (Cs) for each mRNA were subtracted from that of β-actin mRNA and averaged and converted from log-linear to linear terms. The primers in the reaction were used as follows:

Human IL-1β (forward, 5′-AGGCTGCTCTGG GATTC-3′, reverse, 5′-GCCACAACAACTGACGC-3′),

Human IL-6 (forward, 5′-TGTAGTGAGGAACA AGCCAGAG-3′, reverse, 5′-TACATTTGCCGAAAGAG CC-3′),

Human TNF-α (forward, 5′-CTTCTCCTTCCT GATCGTGG-3′, reverse, 5′-GCTGGTTATCTCTCAG CTCCA-3′),

Human β-actin (forward, 5′-CTGTCCCTGTATG CCTCT-3′, reverse, 5′-ATGTCACGCACGATTTCC-3′).

### Preparation of cytosolic and nuclear extracts and whole cell lysates

Nuclear and cytosolic protein extracts were prepared according to the instructions of the kit (KeyGEN, Nanjing, China). One part of the cytosolic and nuclear fractions was subjected to immunoblot analysis. The rest of the nuclear extract was used for electrophoretic mobility-shift assay (EMSA).

The whole cell lysates was isolated by Pierce RIPA buffer added with protease inhibitors (1 mM Phenylmethanesulfonyl fluoride, 0.1 mM dithiothreitol, 0.1 Mm NaF, 0.1 mM Leupeptin), incubated on ice for 50 min to allow cells to swell and then centrifuged at 12,000 rpm for 30 min at 4°C. The supernatants were saved as the whole cell lysates and measured using the BCA protein assay method with Varioskan spectrofluorometer and spectrophotometer (Thermo) at 562 nm.

### Western blot analysis

Protein samples were separated by 10% SDS-PAGE and transferred onto nitrocellulose membranes. The membranes were blocked with 1% BSA at 37°C for 1 h and incubated with indicated antibodies overnight at 4°C, followed by IRDye800 conjugated secondary antibody for 1 h at 37°C. Immunoreactive protein was detected with an Odyssey Scanning System (LI-COR Inc., Lincoln, Nebraska).

### Immunofluorescence microscopy

THP-1 cells were pretreated with LPS (1 μg/ml) and LL202 (0.8 μM) for 6 h and then harvested. Cells were fixed with 4% paraformaldehyde in PBS, permeabilized with 0.5% Triton X-100, and blocked with 3% BSA for 1 h. Samples were incubated with primary antibodies against c-Jun (diluted 1:100)and c-Fos (diluted 1:50) overnight at 4°C. After washed, cells were exposed to FITC-conjugated secondary antibodies (1:1000, Invitrogen, CA, USA). Samples were observed and captured with a confocal laser scanning microscope (Olympus Corp., Tokyo, Japan).

### Electrophoretic mobility shift assays (EMSA)

Nuclear extracts were isolated as described above. EMSA was then performed with a non-radioactive (biotin label) gel shift assay according to the manufacturer's protocol. The AP-1 consensus oligonucleotide probes (5′-CGCTTGATGACTCAGCCGGAA-3′ and 3′-GCG AACTACTGAGTCGGCCTT-5′) were end labeled with biotin using terminal deoxynucleotidyl transferase. The oligonucleotides were annealed to their complementary oligonucleotides and incubated with nucleoproteins for 30 min at 25°C. Samples were run on a 6% polyacrylamide gel, which was transferred into Nylon member and then blocked and washed. Bands were detected by chemiluminescent method as previously described [46].

### Statistical analysis

The data shown in the study were obtained in at least three independent experiments and all results represent the mean ± SD. Differences between the groups were assessed by one-way ANOVA and Dunnett's post hoc test. Details of each statistical analysis used are provided in the figure legends. Differences with *P* values < 0.05 were considered statistically significant.

## Abbreviations

DSS: dextran sulfate sodium
MPO: myeloperoxidase
iNOS: inducible nitric oxide synthase
AP-1: activator protein-1
MAPK: mitogen-activated protein kinases
BSA: bovine serum albumin
BMDM: bone marrow derived macrophages
IBD: inflammatory bowel disease
UC: Ulcerative colitis
DAPI: diamidino-phenyl-indole
DMSO: dimethylsulfoxide
GSH: glutathione
IL-1β: interleukin-1 beta
IL-6: interleukin-6
LPS: lipopolysaccharides
PBS: phosphatebuffered saline
TNF-α: tumor necrosis factor alpha.
